# Cpg boosts peptide-based oral immunotherapy in mice with peanut allergy

**DOI:** 10.3389/falgy.2026.1788819

**Published:** 2026-06-19

**Authors:** Fang Liu, Jun-Hui Yang, Lei Han, Qiao Liu, Yang-Peng Wu, Yang-Dong Wu, Shu-Wang Peng, Xiong-Jun Peng, Ping-Chang Yang, Gui-Xiang Tian

**Affiliations:** 1 Department of Ultrasoud, The First Hospital of Hunan University of Chinese Medicine, Changsha, China; 2 Department of Traditional Chinese Medicine Diagnostics, Hunan University of Chinese Medicine, Changsha, China; 3 Department of Ultrasoud, The Second Xiangya Hospital, Central South University, Changsha, China; 4 Department of Digestive Endoscopy Center, The First Hospital of Hunan University of Chinese Medicine, Changsha, China; 5 Department of Gastrointestinal and Thyroid and Vascular Surgery, The First Hospital of Hunan University of Chinese Medicine, Changsha, China; 6 Department of Equipment Division, The Second Xiangya Hospital, Central South University, Changsha, China; 7 Guangdong Provincial Key Laboratory of Regional Immunity and Diseases, Shenzhen, China; 8 Research Center of Allergy & Immunology, Shenzhen University School of Medicine, Shenzhen, China

**Keywords:** b10 cells, cpG ODN, immunotherapy, liposome, peanut allergy, tr1 cells

## Abstract

**Background:**

Effective therapies for peanut allergy (PA) remain limited. Cytosine-phosphate-guanine oligodeoxynucleotides (CpG ODN) have emerged as promising adjuvants for allergen-specific immunotherapy (AIT). This study aimed to evaluate whether a liposomal delivery system co-encapsulating peanut extract and CpG enhances therapeutic efficacy in a murine PA model and to elucidate the underlying immunoregulatory mechanisms.

**Methods:**

A murine PA model was established using peanut extract. Liposomes were formulated to carry peanut extract alone (LipP), CpG alone (LipC), both (LipCP), or were empty (LipE). PA mice received daily gavage of these formulations for two weeks. Clinical symptoms, antibody profiles, intestinal mediator release, and regulatory immune cell populations were assessed.

**Results:**

PA mice exhibited severe anaphylaxis, including a 6.0-fold increase in diarrhea incidence (Cohen's d = 8.21, 95% CI: 5.14–11.28) and a 2.3 °C median core temperature decrease (Cohen's d = 7.94, 95% CI: 4.89–10.99) compared to naive controls (NC). LipCP treatment substantially reduced PA severity: diarrhea incidence decreased by 89% (Cohen's d = 7.86, 95% CI: 4.78–10.94), core temperature reduction was abolished, serum IgE decreased by 78% (Cohen's d = 6.58, 95% CI: 3.85–9.31), and IgG2a/IgG2c levels were restored. LipCP also suppressed intestinal mast cell and eosinophil activation and increased intestinal B10 cell frequency by 3.1-fold (Cohen's d = 6.27, 95% CI: 3.64–8.90) and Tr1 cell frequency by 4.2-fold (Cohen's d = 7.15, 95% CI: 4.21–10.09). In contrast, LipP, LipC, or LipE showed only modest, non-significant effects.

**Conclusions:**

Liposomal co-delivery of peanut extract and CpG effectively suppresses peanut allergy in mice by restoring B10 cell and Tr1 cell-mediated immune tolerance. This approach warrants further investigation for clinical translation.

## Introduction

Peanut allergy (PA) is an adverse immune response to ingested peanut allergens, ranging from mild gastrointestinal discomfort to life-threatening anaphylaxis (1–3). Currently, patients must strictly avoid peanut consumption, as effective curative therapies are lacking (4–6). PA is characterized by Th2 cell polarization (7–9), leading to IgE production, mast cell sensitization, and subsequent mediator release upon re-exposure (10, 11).

The immune regulatory system, comprising regulatory B cells (Breg) and regulatory T cells (Treg/Tr1), maintains homeostasis by limiting excessive immune responses (12). IL-10-producing B cells (B10 cells) and type 1 regulatory T cells (Tr1 cells) are critical for suppressing Th2-driven inflammation (13, 14). Dysfunction of these populations is linked to food allergy pathogenesis (15–17). Enhancing IL-10 production by B10 or Tr1 cells is therefore a promising therapeutic strategy.

Allergen-specific immunotherapy (AIT) is the only disease-modifying treatment for allergic diseases (18). However, its efficacy and safety require improvement. CpG oligodeoxynucleotides (CpG ODN), unmethylated DNA motifs recognized by Toll-like receptor 9 (TLR9), are potent immune modulators. CpG has been FDA-approved as an adjuvant for hepatitis B vaccines (19) and has shown promise in AIT for allergic rhinitis and asthma (20, 21). CpG shifts immune responses from Th2 toward Th1 and regulatory phenotypes, enhancing tolerance (19). Despite this, the optimal delivery strategy for CpG in PA remains unexplored.

We hypothesized that co-delivering peanut allergen and CpG via liposomes would protect them from gastric degradation, enhance uptake by intestinal immune cells, and synergistically restore tolerance. In this study, we developed LipCP (liposome with peanut extract + CpG) and demonstrated its superior efficacy over liposomes containing either component alone in a murine PA model. LipCP restored B10 and Tr1 cell functions, suppressed mast cells and eosinophils, and alleviated anaphylaxis.

## Materials and methods

### Establishment of a PA murine model

Peanut extracts were prepared in-house (22), and protein content was determined by the Bradford method (23). Male and female C57BL/6 mice were sensitized by subcutaneous injection of peanut extract (0.1 mg/mouse) mixed with alum (0.2 mg) on days 1 and 7 (Supplementary Figure S1). From days 9–22, mice received oral gavage of peanut extract (1 mg/mouse in 0.3 mL PBS) every other day. On day 23, mice were orally challenged with 50 mg peanut extract. Naive control mice received PBS.

### Preparation and characterization of liposomes

**Figure 1:**
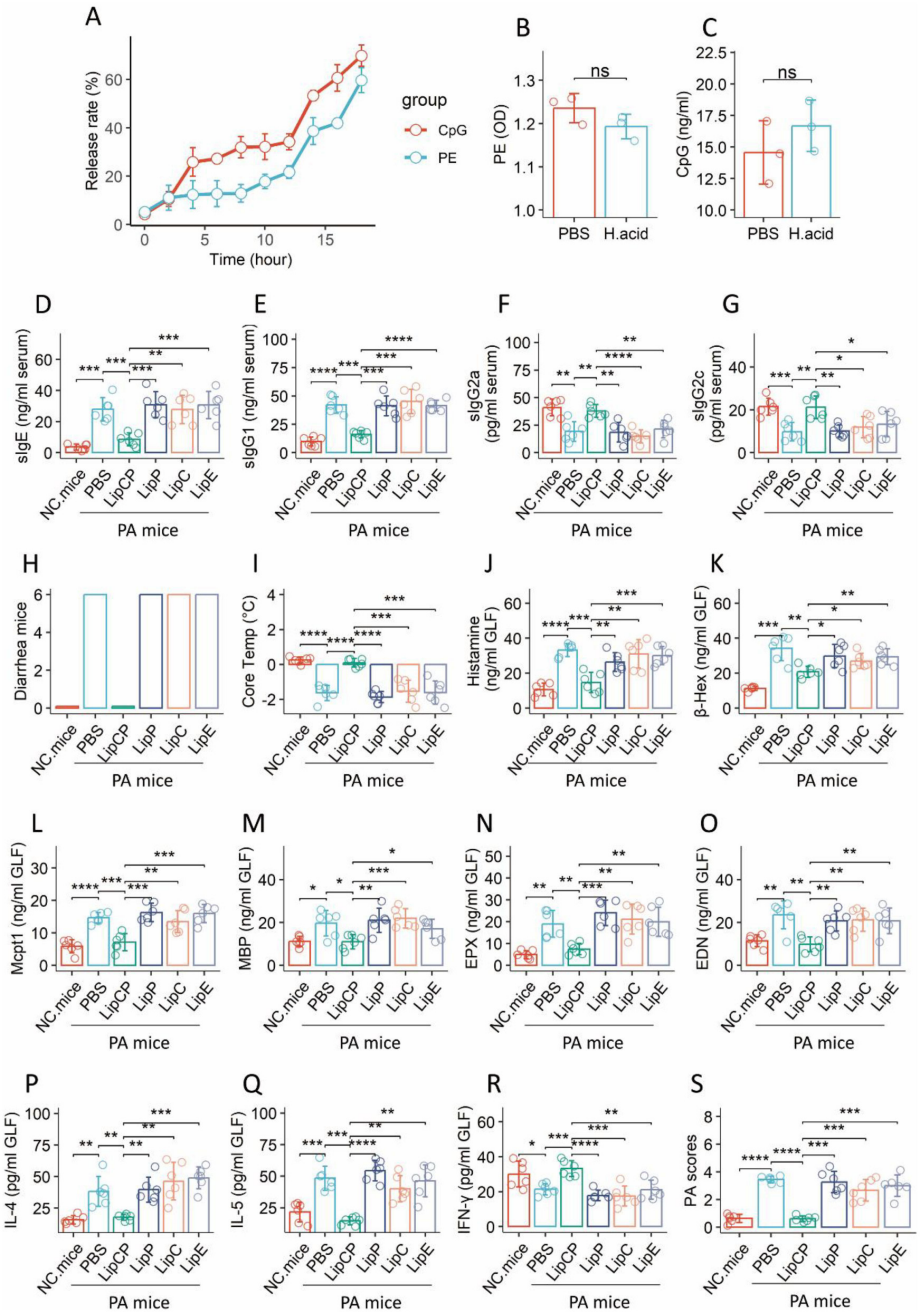
Administration of LipCP suppresses PA response. **A**, line plots show the release rate of CpG and PE from LipCP in an *in vitro* experiment. **B**, the amount of PE (peanut extract). **C**, the amount of CpG. **D–G**, serum levels of sIgE **(D)**, sIgG1 **(E)**, sIgG2a **(F)**, and sIgG2c **(G) H,I**, diarrhea mice **(H)** and decrease in core temperature **(I)** in mice after antigen challenge. **J–R**, the amounts of indicated cytokines in GLF. **S**, PA scores. Each group consists of 6 mice. Each dot in bars presents one sample (assessed in triplicate). The data of bars are mean ± SD. Statistics: ANOVA + Tukey HSD test. *p < 0.05; **p < 0.01; ***p < 0.001; ****p < 0.0001. PA, peanut allergy; NC, naïve control; GLF, gut lavage fluid; LipCP, liposomes containing CpG and peanut; LipP, liposomes containing peanut; LipC, liposomes containing CpG; LipE, empty liposomes; ß-Hex, ß-Hexosaminidase. Each experiment was repeated three times.

Liposomes were formulated by thin-film hydration. LipCP contained peanut extract and CpG ODN 1826 (Class B, 5 µg CpG per dose). LipP contained peanut extract only, LipC contained CpG only, and LipE was empty. Particle size, polydispersity index (PDI), zeta potential, encapsulation efficiency, and storage stability were characterized (Supplementary Table S1, Figures 1A–D). The CpG dose (5 µg/mouse) was selected based on previous AIT optimization studies showing robust Th1/regulatory responses without excessive inflammation (19). *In vitro*release of peanut protein and CpG from LipCP was time-dependent (Figure 1E). Gastric acid (pH 1.0) did not affect peanut protein bioactivity or CpG integrity (Figures 1F,G).

### Administration of liposomes

After the last peanut sensitization (day 22), mice received daily gavage of LipCP, LipP, LipC, or LipE (equivalent to 1 mg peanut protein/mouse in 0.3 mL saline) for two weeks. On day 37, mice were challenged with 5 mg peanut extract, and PA responses were assessed. The 1 mg peanut protein dose is within the optimal range for murine AIT, balancing immunogenicity and safety (20).

### Assessment of PA response

Diarrhea incidence was recorded within 30 min post-challenge. Core temperature was measured rectally. Mice were euthanized, and jejunal segments (15 cm) were lavaged with 1 mL PBS to obtain gut lavage fluid (GLF).

### Flow cytometry (FCM)

Single cells from jejunum (lamina propria mononuclear cells, LPMCs) were stained with fluorophore-conjugated antibodies (Supplementary Table S2) for surface and intracellular markers (B10 cells: CD19⁺CD1d⁺CD5⁺; Tr1 cells: CD4⁺LAG3⁺CD49b⁺) as described (16, 24, 25). Data were acquired on a BD FACSCanto II and analyzed with FlowJo.

### Purification of B10 and Tr1 cells

LPMCs were sorted using a BD Aria flow cytometer to isolate CD19⁺CD1d⁺CD5⁺ B10 cells and CD4⁺LAG3⁺CD49b⁺ Tr1 cells (purity >90%; Supplementary Figure S2).

### IL-10 promoter methylation analysis

Genomic DNA from B10 cells was bisulfite-treated (EZ DNA Methylation Kit, Zymo Research). Methylation-specific PCR amplified unmethylated and methylated regions of the *Il10*promoter using primers: unmethylated (forward: 5′-gaggttttgaagaaaattagttttttt-3′, reverse: 5′-aaaccctcatctataaaattccattc-3′); methylated (forward: 5′-gtttgaagaaaaattagtttttttgg-3′, reverse: 5′-cctccactcaacctaaaaattaaacat-3′).

### Enzyme-Linked immunosorbent assay (ELISA)

Serum and GLF levels of IgE, IgG1, IgG2a, IgG2c, histamine, *β*-hexosaminidase, mouse mast cell protease-1 (Mcpt1), eosinophil peroxidase (EPX), major basic protein (MBP), and cytokines (IL-4, IL-5, IL-13, IFN-*γ*, IL-10) were measured by ELISA kits.

### Statistical analysis

Continuous outcomes were compared by Student's *t*-test (two groups) or one-way ANOVA with Tukey's *post-hoc* test (multiple groups). Categorical outcomes (diarrhea incidence) used Fisher's exact test or logistic regression. Effect sizes were Cohen's *d*(continuous) or odds ratios (OR) with 95% confidence intervals (CIs). *p* < 0.05 was significant. Analyses used GraphPad Prism 9.0 and R v4.3.1.

## Results

### Liposomal formulations and stability

LipCP exhibited a uniform size (∼150 nm, PDI≈0.2) and negative zeta potential (−20 mV), indicating stable, monodisperse liposomes (Figures 1A–D). Encapsulation efficiency exceeded 85% for both peanut extract and CpG. Storage at 4 °C maintained stability for 28 days. *In vitro*release showed sustained delivery over 48 h. Gastric acid (pH 1.0) did not degrade peanut proteins or CpG (Figures 1F, G).

### LipCP substantially reduces PA severity

**Figure 2:**
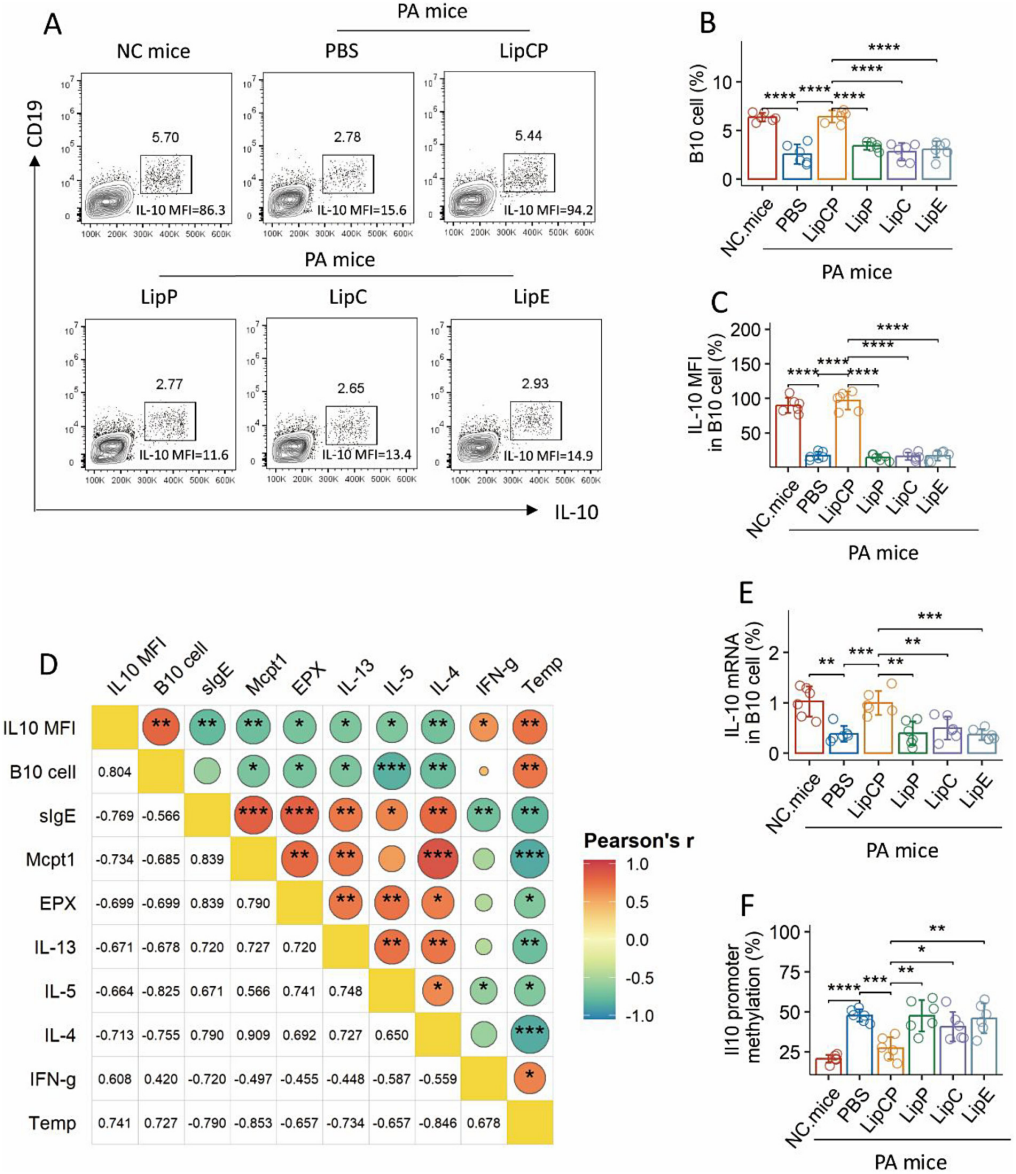
Assessment of correlation between intestinal B10 cells and PA response. **A**, gated FCM plots show B10 cells. **B**, B10 cell counts. **C**, IL-10 MFI in B10 cells. **D**, correlation coefficients between B10 cell counts/IL-10 MFI and the parameters of PA response (data are presented in Figure 1). **E,F**, IL-10 mRNA **(E)** and IL-10 promoter methylation **(F)** in B10 cells. The data are presented as mean ± SD. Each dot in bars presents one sample (assessed in triplicate). Statistics: ANOVA + Tukey HSD test **(B,C,E,F)** and Pearson correlation coefficient test **(D)** *p < 0.05; **p < 0.01; ***p < 0.001; ****p < 0.0001. Each group consists of 6 mice. The experiments were repeated 3 times. Temp: Changes of core temperature.

PA mice showed severe anaphylaxis vs. NC: 6.0-fold higher diarrhea incidence (Cohen's d = 8.21, *p* < 0.0001), 2.3 °C core temperature drop (Cohen's d = 7.94, *p* < 0.0001), 3.8-fold higher serum IgE (Cohen's d = 6.73, *p* < 0.0001), 2.1-fold higher IgG1 (Cohen's d = 5.82, *p* < 0.0001), and 72%/68% lower IgG2a/IgG2c (Cohen's d ≥ 5.97, *p* < 0.0001) (Figures 2A–D). GLF from PA mice had 2.5–4.0-fold increases in allergic mediators (histamine, *β*-hexosaminidase, Mcpt1, EPX, MBP) and Th2 cytokines (IL-4, IL-5, IL-13), with 75% lower IFN-*γ* (all *p* < 0.0001) (Figures 2E–P).

LipCP treatment markedly attenuated all PA abnormalities (*p* < 0.0001): diarrhea incidence decreased by 89% (Cohen's d = 7.86), core temperature normalized, serum IgE decreased by 78% (Cohen's d = 6.58), IgG1 decreased by 65% (Cohen's d = 5.71), IgG2a/IgG2c increased by 70%/66% (Cohen's d ≥ 5.84), and GLF mediators/cytokines decreased by 60%–85% (Cohen's d ≥ 3.95) (Figure 2). In contrast, LipP, LipC, or LipE produced only modest, non-significant changes (all *p* > 0.05, Cohen's d < 1.20).

### LipCP promotes IL-10 expression in B10 cells

**Figure 3:**
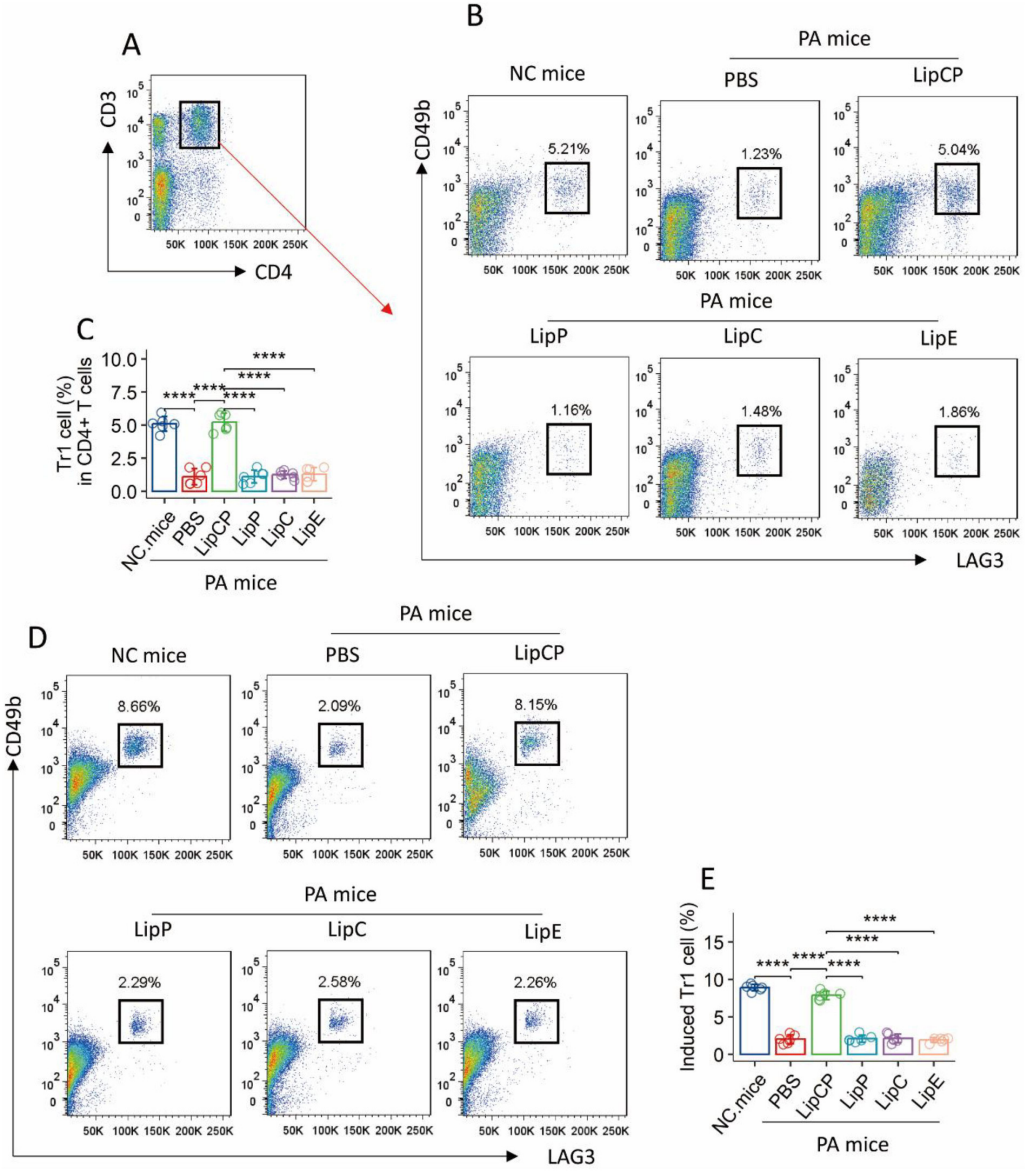
LipCP restores the immune tolerogenic properties of B10 cells. **A**, CD4^+^ T cells were gated from LPMCs. **B**, gated plots show Tr1 cells in CD4^+^ T cells. **C**, bars show mean ± SD of Tr1 cells in LPMCs. **D,E**, gated plots show induced Tr1 cells; bars indicate mean ± SD of induced Tr1 cells. The data of **A,B,D** are from one experiment that represent three independent experiments. Each dot in bars presents one sample (tested in triplicate). Statistics: ANOVA + Tukey HSD test. ****p < 0.0001. Each experiment was repeated three times.

PA mice had 71% lower intestinal B10 cell frequency (Cohen's d = 6.35, *p* < 0.0001) and 79% lower IL-10 MFI (Cohen's d = 7.02, *p* < 0.0001) vs. NC (Figures 3A–C). *Il10*mRNA was 82% lower (Cohen's d = 6.89, *p* < 0.0001), and *Il10*promoter methylation was 3.9-fold higher (Cohen's d = 6.47, *p* < 0.0001) in PA B10 cells (Figures 3D, E). LipCP increased B10 frequency by 3.1-fold (Cohen's d = 6.27, *p* < 0.0001) and restored IL-10 production, while LipP, LipC, and LipE were ineffective (Figure 3).

### LipCP enhances immune tolerogenic functions of B10 cells

**Figure 4:**
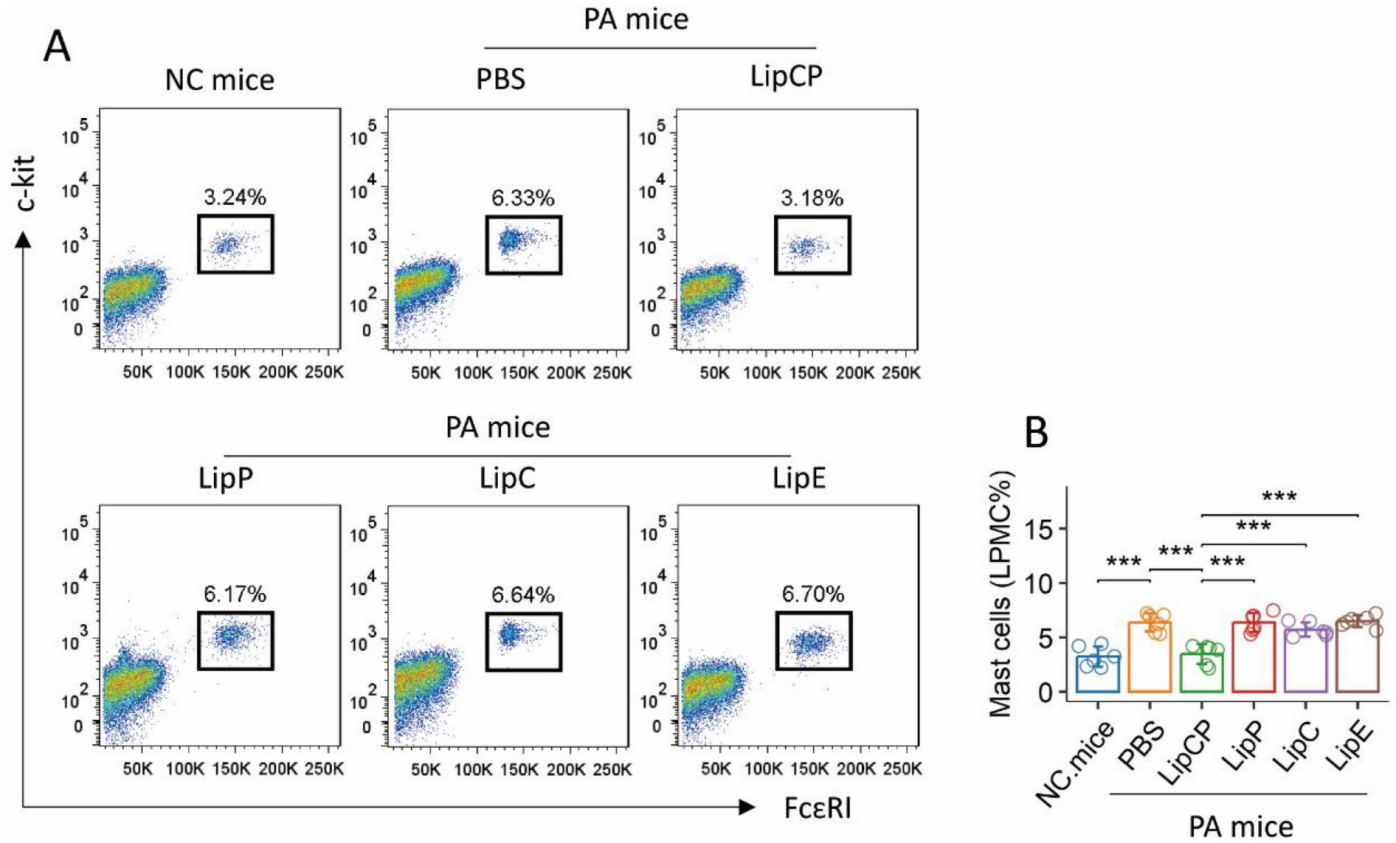
LipCP suppresses mast cells in the intestine of PA mice. **A**, gated FCM plots show mast cells. **B**, bars show mean ± SD of mast cell counts in LPMCs of 6 mice per group. Each dot in bars presents one sample (assessed in triplicate). Statistics: ANOVA + Tukey HSD test. ***p < 0.001. The data of panel A are from one experiment that represents three independent experiments.

LipCP increased intestinal Tr1 cell frequency by 4.2-fold (Cohen's d = 7.15, *p* < 0.0001) (Figures 4A–C). Co-culture of naïve CD4⁺ T cells with LipCP-primed B10 cells induced ∼5% Tr1 cells (2.5-fold higher than LipP/LipC-primed B10; *p* < 0.0001) (Figures 4D, E). LipP, LipC, and LipE did not increase Tr1 cells (Figure 4).

### LipCP suppresses mast cells and eosinophils in the intestine

**Figure 5:**
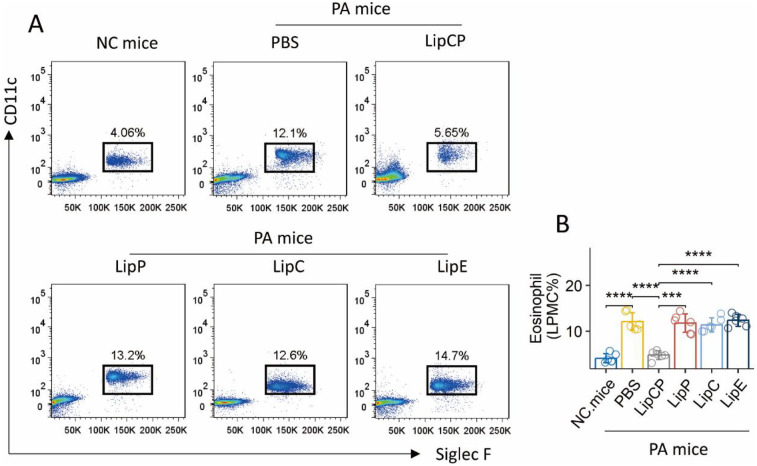
LipCP suppresses eosinophils in the intestine of PA mice. **A**, gated FCM plots show eosinophils. **B**, bars show mean ± SD of eosinophil counts in LPMCs of 6 mice per group. Each dot in bars presents one sample (assessed in triplicate). Statistics: ANOVA + Tukey HSD test. ***p < 0.001; ****p < 0.0001. The data of panel A are from one experiment that represents three independent experiments.

PA mice had 6.38 ± 0.83% mast cells in LPMCs vs. 3.23 ± 0.93% in NC. LipCP reduced mast cell frequency by 46% to 3.47 ± 0.85% (*p* < 0.001), comparable to NC. LipP, LipC, and LipE had no effect (Figures 5A–C). GLF histamine and Mcpt1 were 3.2-fold and 4.0-fold higher in PA mice (*p* < 0.0001) and decreased by 79% and 82% with LipCP (*p* < 0.0001) (Figures 5D, E).

**Figure 6:**
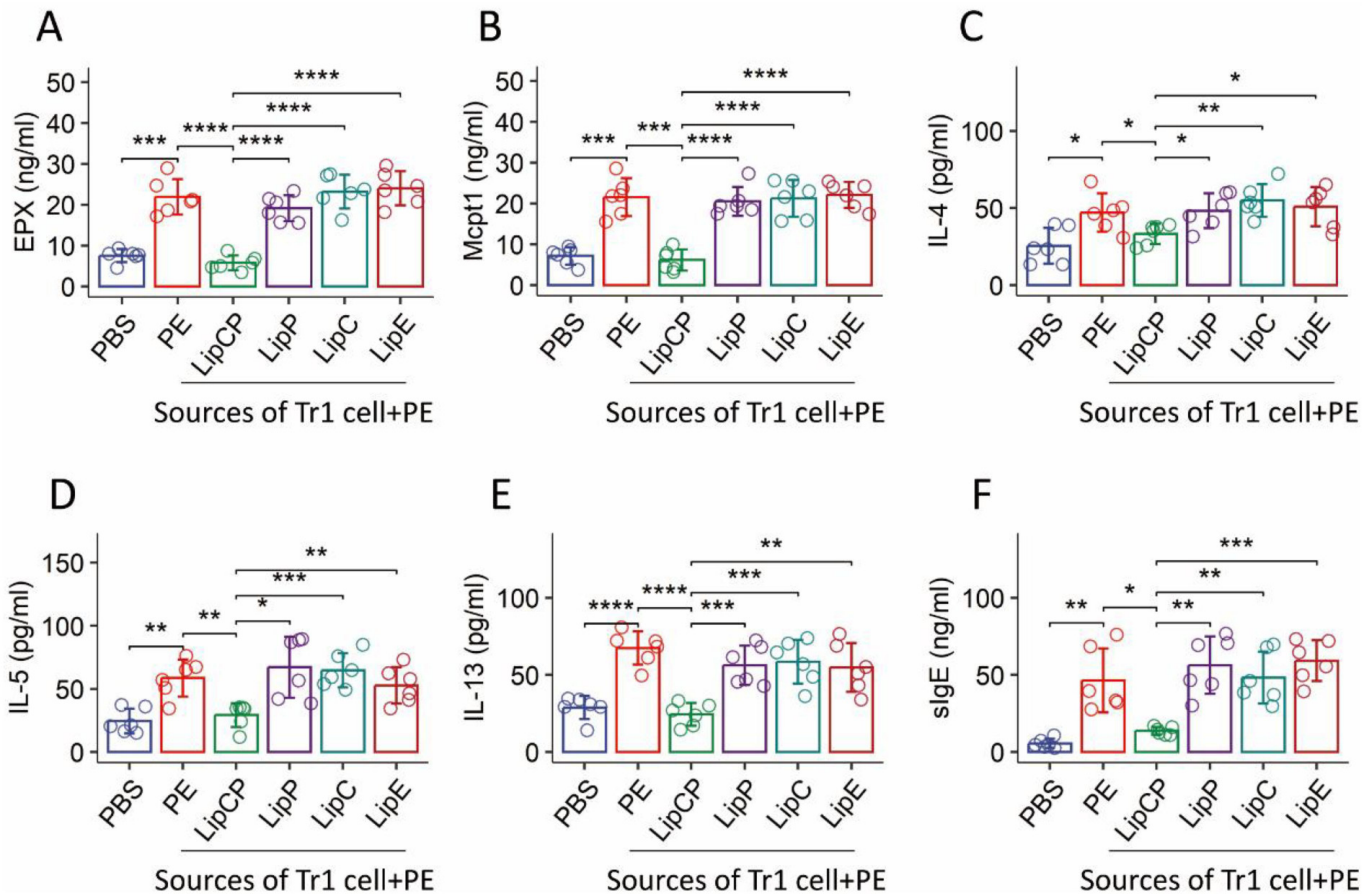
Assessment of immune suppressive functions of Tr1 cells. CD4^+^ T cells and Tr1 cells were isolated from the mouse intestinal tissues after the treatment with the items listed on the *X* axis of bar graphs. CD4^+^ T cells (10^6^/sample) and Tr1 cells (10^5^/sample) were cocultured in the presence of PE (1 μg/mL) and dendritic cells (10^5^/sample) for 24 h. A-F, bars exhibit the amounts (mean ± SD) of indicated molecules in supernatant (by ELISA) from 6 mice per group. Each dot in bars presents one sample (tested in triplicate). Statistics: ANOVA + Tukey HSD test. *p < 0.05; **p < 0.01; ***p < 0.001; ****p < 0.0001. The experiments were repeated three times. PE, Peanut extracts.

Eosinophil frequency was 12.08 ± 1.93% in PA mice vs. 4.07 ± 0.99% in NC. LipCP reduced eosinophils by 61% to 4.79 ± 0.94% (*p* < 0.0001), comparable to NC. LipP, LipC, and LipE were ineffective (Figures 6A–C). GLF EPX and MBP were 3.5-fold and 3.1-fold higher in PA mice (*p* < 0.0001) and decreased by 75% and 71% with LipCP (*p* < 0.0001) (Figures 6D, E).

### Intestinal Tr1 cells from LipCP-treated mice show robust immune suppressive functions

**Figure 7:**
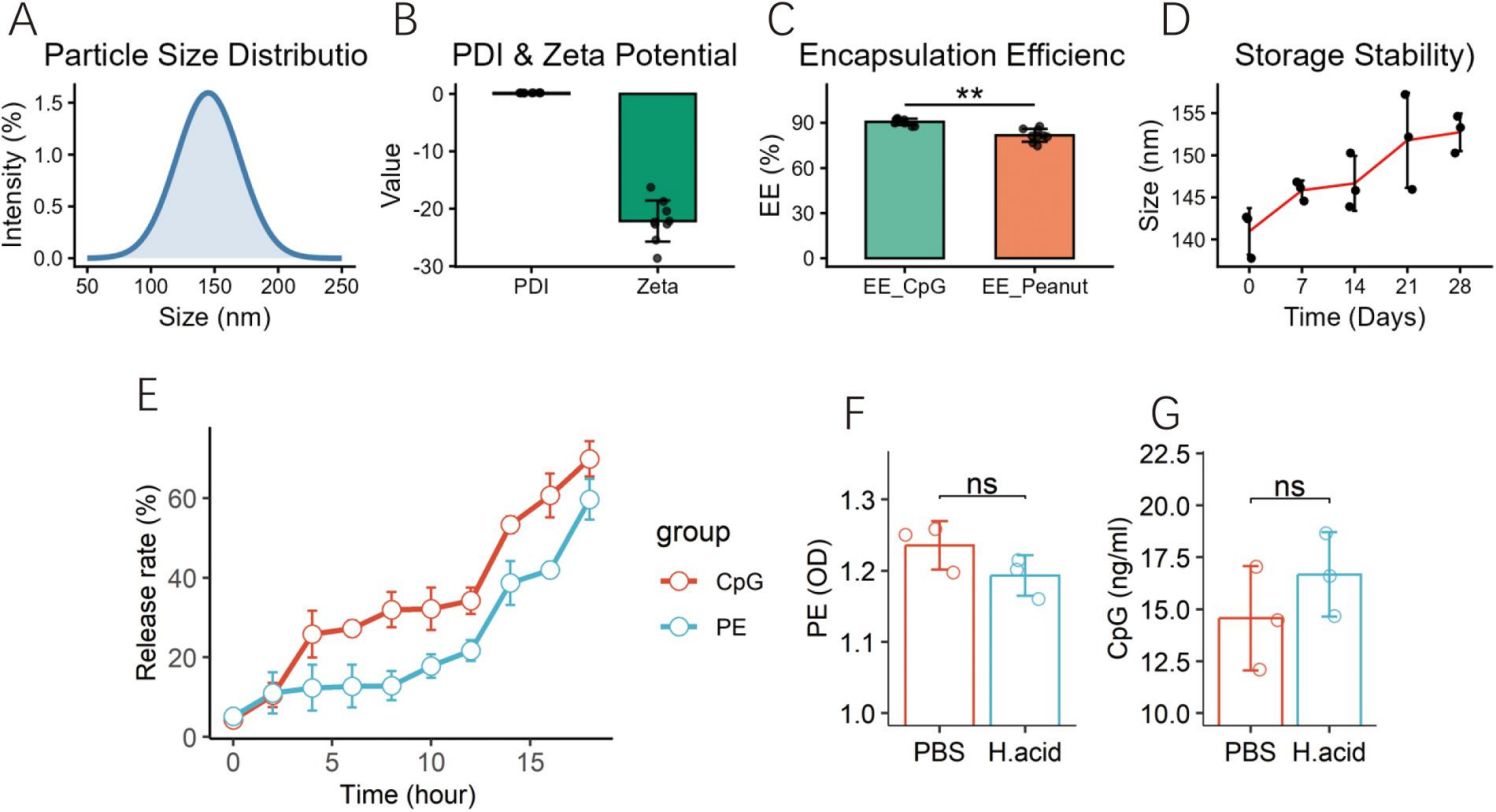
Characterization of CpG and Peanut liposomal formulations. Physicochemical and functional characterization assays were performed on liposomal formulations co-encapsulating CpG and peanut antigen (LipCP) to validate formulation quality, antigen encapsulation, storage stability, and in vitro release kinetics. **A**, particle size distribution of the liposomes, showing a single, narrow peak centered at approximately 150 nm. **B**, polydispersity index (PDI) and zeta potential of the liposomes, with PDI values near 0 indicating a monodisperse population and zeta potential values around -20 mV. **C**, encapsulation efficiency (EE) of CpG and Peanut antigens within the liposomes, with no statistically significant difference observed between the two formulations. **D**, storage stability of the liposomes at 4°C, as indicated by particle size measurements over 28 days, showing minimal variation and confirming formulation stability. **E**, line plots show the release rate of CpG and PE from LipCP in an *in vitro* experiment. **F**, the amount of PE (peanut extract). **G**, the amount of CpG. ns, not significant (*t* test).

Tr1 cells from PA mice failed to suppress mediator release from effector immune cells (EICs) upon peanut extract challenge. Tr1 cells from LipCP-treated mice suppressed EPX, Mcpt1, IL-4, IL-5, IL-13, and sIgE release by 75%–80% (*p* < 0.0001) (Figure 7). Tr1 cells from LipP-, LipC-, or LipE-treated mice showed no suppression.

## Discussion

This study demonstrates that liposomal co-delivery of peanut extract and CpG (LipCP) effectively suppresses peanut allergy in mice. LipCP outperformed liposomes containing either component alone, reducing anaphylaxis, shifting antibody profiles toward Th1/regulatory phenotypes, and restoring B10 and Tr1 cell functions. These findings align with reports that CpG enhances AIT efficacy in allergic rhinitis and asthma (19, 20). The FDA approval of CpG as a hepatitis B vaccine adjuvant underscores its clinical safety and utility (19).

Why was LipCP superior to LipC (CpG alone)? We propose three mechanisms: (1) Protection from degradation: Liposomes shielded CpG and peanut antigen from gastric acid (pH 1.0), ensuring bioactive delivery to intestinal immune cells. (2) Enhanced targeting: The ∼150 nm size favors uptake by dendritic cells and macrophages in gut-associated lymphoid tissue. (3) Synergy: Co-encapsulation delivers antigen and adjuvant to the same antigen-presenting cells, promoting tolerogenic programming (19). In contrast, free CpG (LipC) may be degraded in the stomach or fail to co-localize with antigen in immune cells, reducing efficacy.

Our data reveal a novel B10→Tr1 regulator*y* axis in peanut allergy. B10 cells from LipCP-treated mice exhibited demethylation of the *Il10*promoter, increased IL-10 production, and the ability to induce Tr1 cells. Tr1 cells, in turn, potently suppressed mast cell and eosinophil activation. This complements the established role of tolerogenic dendritic cells in Tr1 induction (26), forming a multilayered regulatory network.

Limitations include the murine model; human responses may differ. Future studies should validate LipCP in humanized models or clinical trials. Nevertheless, LipCP represents a promising, translatable platform for peanut allergy and potentially other food allergies.

## Data Availability

The original contributions presented in the study are included in the article/Supplementary Material, further inquiries can be directed to the corresponding author.

## References

[1] Moneret-Vautrin DA Rance F Kanny G Olsewski A Gueant JL Dutau G. Food allergy to peanuts in France–evaluation of 142 observations. Clin Exp Allergy. (1998) 28(9):1113–9. 10.1046/j.1365-2222.1998.00370.x 9761015

[2] Khodoun M Strait R Orekov T Hogan S Karasuyama H Herbert DR. Peanuts can contribute to anaphylactic shock by activating complement. J Allergy Clin Immunol. (2009) 123(2):342–51. 10.1016/j.jaci.2008.11.004 19121857 PMC2670761

[3] Perkin MR Togias A Koplin J Sicherer S. Food allergy prevention: more than peanut. J Allergy Clin Immunol Pract. (2020) 8(1):1–13. 10.1016/j.jaip.2019.11.002 31950900

[4] Du Toit G Roberts G Sayre PH Bahnson HT Radulovic S Santos AF. Randomized trial of peanut consumption in infants at risk for peanut allergy. N Engl J Med. (2015) 372(9):803–13. 10.1056/NEJMoa1414850 25705822 PMC4416404

[5] Kim EH Yang L Ye P Guo R Li Q Kulis MD. Long-term sublingual immunotherapy for peanut allergy in children: clinical and immunologic evidence of desensitization. J Allergy Clin Immunol. (2019) 144(5):1320–6. 10.1016/j.jaci.2019.07.030 31493887 PMC6842439

[6] Jones SM Kim EH Nadeau KC Nowak-Wegrzyn A Wood RA Sampson HA. Efficacy and safety of oral immunotherapy in children aged 1–3 years with peanut allergy (the immune tolerance network IMPACT trial): a randomised placebo-controlled study. Lancet. (2022) 399(10322):359–71. 10.1016/S0140-6736(21)02390-4 35065784 PMC9119642

[7] Barshow SM Kulis MD Burks AW Kim EH. Mechanisms of oral immunotherapy. Clin Exp Allergy. (2021) 51(4):527–35. 10.1111/cea.13824 33417257 PMC9362513

[8] Hammad H Lambrecht BN. The basic immunology of asthma. Cell. (2021) 184(6):1469–85. 10.1016/j.cell.2021.02.016 33711259

[9] Ogulur I Pat Y Ardicli O Barletta E Cevhertas L Fernandez-Santamaria R. Advances and highlights in biomarkers of allergic diseases. Allergy. (2021) 76(12):3659–86. 10.1111/all.15089 34519063 PMC9292545

[10] Ganeshan K Neilsen CV Hadsaitong A Schleimer RP Luo X Bryce PJ. Impairing oral tolerance promotes allergy and anaphylaxis: a new murine food allergy model. J Allergy Clin Immunol. (2009) 123(1):231–8. 10.1016/j.jaci.2008.10.011 19022495 PMC2787105

[11] Kanagaratham C El Ansari YS Lewis OL Oettgen HC. Ige and IgG antibodies as regulators of mast cell and basophil functions in food allergy. Front Immunol. (2020) 11:603050. 10.3389/fimmu.2020.603050 33362785 PMC7759531

[12] Satitsuksanoa P Jansen K Głobińska A van de Veen W Akdis M. Regulatory immune mechanisms in tolerance to food allergy. Front Immunol. (2018) 9:2939. 10.3389/fimmu.2018.02939 30619299 PMC6299021

[13] Rubtsov YP Rasmussen JP Chi EY Fontenot J Castelli L Ye X. Regulatory T cell-derived interleukin-10 limits inflammation at environmental interfaces. Immunity. (2008) 28(4):546–58. 10.1016/j.immuni.2008.02.017 18387831

[14] Rosser EC Mauri C. Regulatory B cells: origin, phenotype, and function. Immunity. (2015) 42(4):607–12. 10.1016/j.immuni.2015.04.005 25902480

[15] Bunyavanich S Berin MC. Food allergy and the microbiome: current understandings and future directions. J Allergy Clin Immunol. (2019) 144(6):1468–77. 10.1016/j.jaci.2019.10.019 31812181 PMC6905201

[16] Gao P Song S Wang Y Liu H Wang X Shu Q. Semaphorin 3 a restores the ability of type 1 regulatory T cells to suppress food allergy. Immunol Res. (2024) 72(2):320–30. 10.1007/s12026-023-09437-6 37999823

[17] Li L Pang W Xu L Zhang Y Zhang H Zhu L. Inhibition of DNMT1 attenuates experimental food allergy. Mol Immunol. (2024) 173:71–9. 10.1016/j.molimm.2024.07.009 39067087

[18] Durham SR Shamji MH. Allergen immunotherapy: past, present and future. Nat Rev Immunol. (2023) 23(5):317–28. 10.1038/s41577-022-00786-1 36253555 PMC9575636

[19] Montamat G Leonard C Poli A Klimek L Ollert M. Cpg adjuvant in allergen-specific immunotherapy: finding the sweet spot for the induction of immune tolerance. Front Immunol. (2021) 12:590054. 10.3389/fimmu.2021.590054 33708195 PMC7940844

[20] Johnson-Weaver BT McRitchie S Mercier KA Pathmasiri W Sumner SJ Chan C. Effect of endotoxin and alum adjuvant vaccine on peanut allergy. J Allergy Clin Immunol. (2018) 141(2):791–4. 10.1016/j.jaci.2017.07.043 28927819 PMC5803373

[21] Johnson-Weaver BT Staats HF Burks AW Kulis MD. Adjuvanted immunotherapy approaches for peanut allergy. Front Immunol. (2018) 9:2156. 10.3389/fimmu.2018.02156 30319619 PMC6167456

[22] Feng BS Chen X He SH Zheng PY Foster J Xing Z. Disruption of T-cell immunoglobulin and mucin domain molecule (TIM)-1/TIM4 interaction as a therapeutic strategy in a dendritic cell-induced peanut allergy model. J Allergy Clin Immunol. (2008) 122(1):55–61. 10.1016/j.jaci.2008.04.036 18547633

[23] Karimi F Hamidian Y Behrouzifar F Mostafazadeh R Ghorbani-HasanSaraei A Alizadeh M. An applicable method for extraction of whole seeds protein and its determination through bradford’s method. Food Chem Toxicol. (2022) 164:113053. 10.1016/j.fct.2022.113053 35460823

[24] Tian GX Peng KP Yu Y Liang CB Xie HQ Guo YY. Propionic acid regulates immune tolerant properties in B cells. J Cell Mol Med. (2022) 26(10):2766–76. 10.1111/jcmm.17287 35343043 PMC9097846

[25] Xue J Suo L An Y Wang X Zhang S Liu H. Phosphatidylserine promotes immunotherapy for airway allergy. Immunol Lett. (2023) 264:46–55. 10.1016/j.imlet.2023.11.006 38008186

[26] Liu M Thijssen S Hennink WE Garssen J van Nostrum CF Willemsen LEM. Oral pretreatment with ß-lactoglobulin derived peptide and CpG co-encapsulated in PLGA nanoparticles prior to sensitizations attenuates cow’s milk allergy development in mice. Front Immunol. (2022) 13:1053107. 10.3389/fimmu.2022.1053107 36703973 PMC9872660

