# Supplementary material for: Cpg boosts peptide-based oral immunotherapy in mice with peanut allergy
Source: Front Allergy. 2026 Jun 19;7:1788819. doi: 10.3389/falgy.2026.1788819 (PMC13329794; doi:10.3389/falgy.2026.1788819)
Supplement: Supplementary file 1 [file Supplementaryfile1.docx]

**Supplemental Materials**

**Reagents**

Antibodies (Abs) of CD3 (Cat#: sc-20047, Clone#: PC3/188A, Alexander Flour (AF): AF546), CD19 (sc-373897, F-3, AF594), IL-10 (sc-365858, A-2, AF648), MHC II (sc-53726, 11-5.2, AF700), CD1d (sc-373858, G-12, AF700), CD5 (sc-1180, UCH-T2, AF648), CD4 (sc-19641, MT310, AF488), LAG3 (sc-514993, D-8, AF594), CD49b (sc-74466, HAS-4, AF647), c-kit (sc-365504, E-3, AF546) and CD11c (sc-398708, 3H986, AF488) were purchased from Santa Cruz Biotech (Santa Cruz, CA). BD OptiBuild™ BV650 Rat Anti-Mouse CD14, Alexa Fluor® 488 Rat Anti-Mouse Siglec-F 567005 and BD OptiBuild™ RY610 Hamster Anti-Mouse FcεR1α (BD Bioscience). ELISA kits and manufacturers: major basic protein (MBP), eosinophil derived neurotoxin (EDN), eosinophil peroxidase (EPX): Kemiao Biotech (Wenzhou, China); histamine (Novus Biologicals); β-Hexosaminidase (Gudou Biotech, Shanghai, China); mouse mast cell protease-1 (Mcpt1; MultiSciences, Hangzhou, China); IL-4, IL-5, IL-13, IFN-γ (R&D Systems). CpG-ODN 1826 (TCCATGACGTTCCTGACGTT) (MedChemExpress, China). Methylation specific PCR kit (Zeye Biotech, Shanghai, China). Materials for liposome construction were purchased from Sigma Aldrich (St. Louis., MO). The peanut-specific IgE (sIgE), sIgG1, sIgG2a and sIgG2c ELISA kits were customizedly made by NeoBioscience (Shenzhen, China).

**Mice**

Specific pathogen-free (SPF) BALB/c mice (a strain widely used in allergy research due to its inherent Th2-biased immune response) were purchased from Beijing Experimental Center (Beijing, China) and used for all experiments. Mice were stratified by sex (female) and age (6–8 weeks old) at the time of study initiation, with an average body weight of 18–22 g to ensure consistent baseline immune status across groups.

Mice were randomly assigned to experimental groups using a computer-generated randomization schedule, with 6 mice per group (group assignments: Control, Peanut Allergy [PA] Model, PA + CpG-Adjuvanted Immunotherapy, and PA + Vehicle-Only Immunotherapy; sample size was determined via power analysis based on preliminary data showing a minimum detectable difference in serum IgE levels of 30% with 80% power and α = 0.05). No mice were excluded from the final analysis, as no animals exhibited unexpected mortality, severe illness, or failure to meet model induction criteria.

All mice were housed in the SPF Animal Facility of Central Southern University under standardized conditions: temperature (22 ± 2 °C), relative humidity (50 ± 10%), and a 12 h light/12 h dark cycle (lights on at 07:00, lights off at 19:00). Mice were housed in polycarbonate cages with autoclaved corn cob bedding and provided ad libitum access to sterile standard rodent chow (free of peanut-derived ingredients) and filtered water. Cages were changed weekly to maintain hygiene, and all handling and experimental procedures were performed in compliance with the Guide for the Care and Use of Laboratory Animals (National Institutes of Health, USA) and approved by the Institutional Animal Care and Use Committee (IACUC) at The Second Xiangya Hospital of Central Southern University (Approve#: A2021034).

Male BALB/c mice were purchased from Beijing Experimental Animal Center (Beijing, China). Mice were maintained in a specific pathogen free facility at Central Southern University following the ARRIVA guideline. Mice were allowed to have free access to water and food. The animal experimental protocol was approved by the Animal Ethics Committee at The Second Xiangya Hospital of Central Southern University (Approve#: A2021034).

**Enzyme-linked immunosorbent assay (ELISA)**

#### The amounts of cytokines and proteins of interest in GLF, or culture supernatant, and serum were determined by ELISA. Commercial reagent kits were purchased to conduct ELISA based on the protocols provided by manufacturers.

#### Sample Preparation

Samples were collected at specified time points and processed immediately. Serum, plasma, or tissue supernatant samples (depending on the analyte of interest) were stored at -80°C until analysis. On the day of the assay, samples were thawed on ice and diluted appropriately with the provided assay buffer or diluent (as specified in the kit instructions). If necessary, samples were centrifuged at 10,000 × g for 5 minutes to remove any debris or particulates before being loaded onto the ELISA plate.

#### ELISA Procedure

1. **Standards and Samples**:
   - Standard curves were prepared using the serial dilutions of the provided reference standards. Each standard was added to duplicate wells, and samples were loaded in triplicate to ensure reliability and minimize variability.
   - The samples were added to the wells, and the plate was gently tapped to ensure even distribution.
2. **Incubation**:
   - The plate was incubated at room temperature (20–25°C) for the time specified in the kit instructions, typically ranging from 1 to 2 hours, depending on the analyte.
3. **Washing**:
   - After incubation, the wells were washed 3–5 times with the provided wash buffer to remove unbound substances. Each wash cycle involved soaking the wells with 300–400 μL of wash buffer and letting it sit for 30 seconds to 1 minute before discarding.
4. **Detection and Signal Amplification**:
   - The conjugate (HRP-conjugated antibody) was added to each well, and the plate was incubated for an additional 30 minutes to 1 hour at room temperature.
   - After a final wash, the chromogenic substrate (TMB) was added to each well. The plate was incubated in the dark for 10–30 minutes, or until the color developed sufficiently.
5. **Stop Reaction**:
   - The reaction was stopped by adding the stop solution (e.g., 1N H_2_SO_4_) to each well. The color change was immediately visible, and the plate was read within 10 minutes to prevent color fading.
6. **Measurement**:
   - The optical density (OD) was measured at the specified wavelength (e.g., 450 nm) using a microplate reader. Background absorbance (from blank wells) was subtracted from all wells.

#### Data Analysis

The concentration of each analyte in the samples was calculated using the standard curve (generated from the serially diluted standards) and the corresponding software provided by the manufacturer or a suitable curve-fitting software. Each sample was analyzed in triplicate, and the mean value was used for further analysis.

**Preparation of lamina propria mononuclear cells (LPMC)**

The small intestine was excised and opened longitudinally. Rinsing with PBS was used to wash out the contents of the intestinal cavity. The tissues were cut into about 5 cm in size and incubated with 0.25% EDTA for 5 minutes. The tissues were cut into small pieces after being washed with PBS. Samples were incubated with collagenase IV (0.5 mg/ml) and DNase I (200 ng/ml) for 30 minutes at 37°C with mild agitation. Single cells were filtered through cell strainers (100 μm first, then 70 μm). LPMCs were isolated from single cells by gradient density Percoll centrifugation.

**Cell culture**

RPMI1640 was used to culture cells. 10 % fetal calf serum, 100 U/ml penicillin, 0.1 mg/ml streptomycin, and 2 mM L-glutamine were added to the medium. Cell viability was over 99% as checked with a Trypan blue exclusion assay.


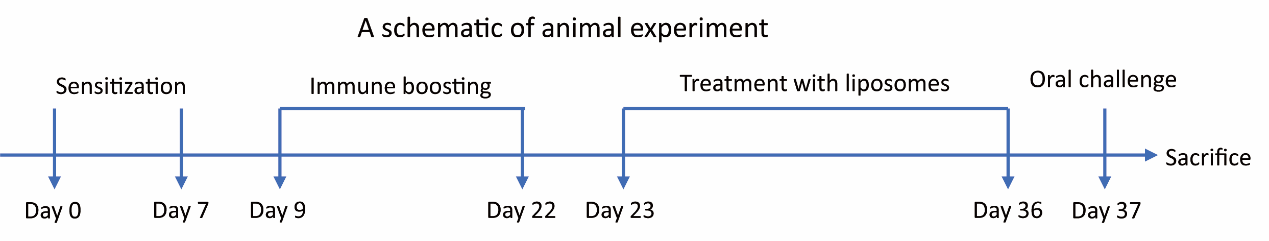


**Figure S1. A schematic of animal experiment**.

**Sensitization**: Mice were sensitized to peanuts by subcutaneous injection with peanut extract (0.1 mg/mouse mixed with 0.2 mg alum) on day 1 and day 7, respectively.

**Immune boosting**: The mice were then received gavage-feeding with peanut extract (1 mg/mouse in 0.3 ml PBS) every other day from day 9 to day 22.

**Treatment with liposomes**: mice received gavage feeding with LipCP, or LipC, or LipP, or LipE (at a dose equaling to 1 mg peanut protein/mouse; in 0.3 ml saline) daily for two weeks.

**Oral challenge**: On day 23, mice received an oral challenge of peanut extract at 50 mg/mouse via gavage-feeding.


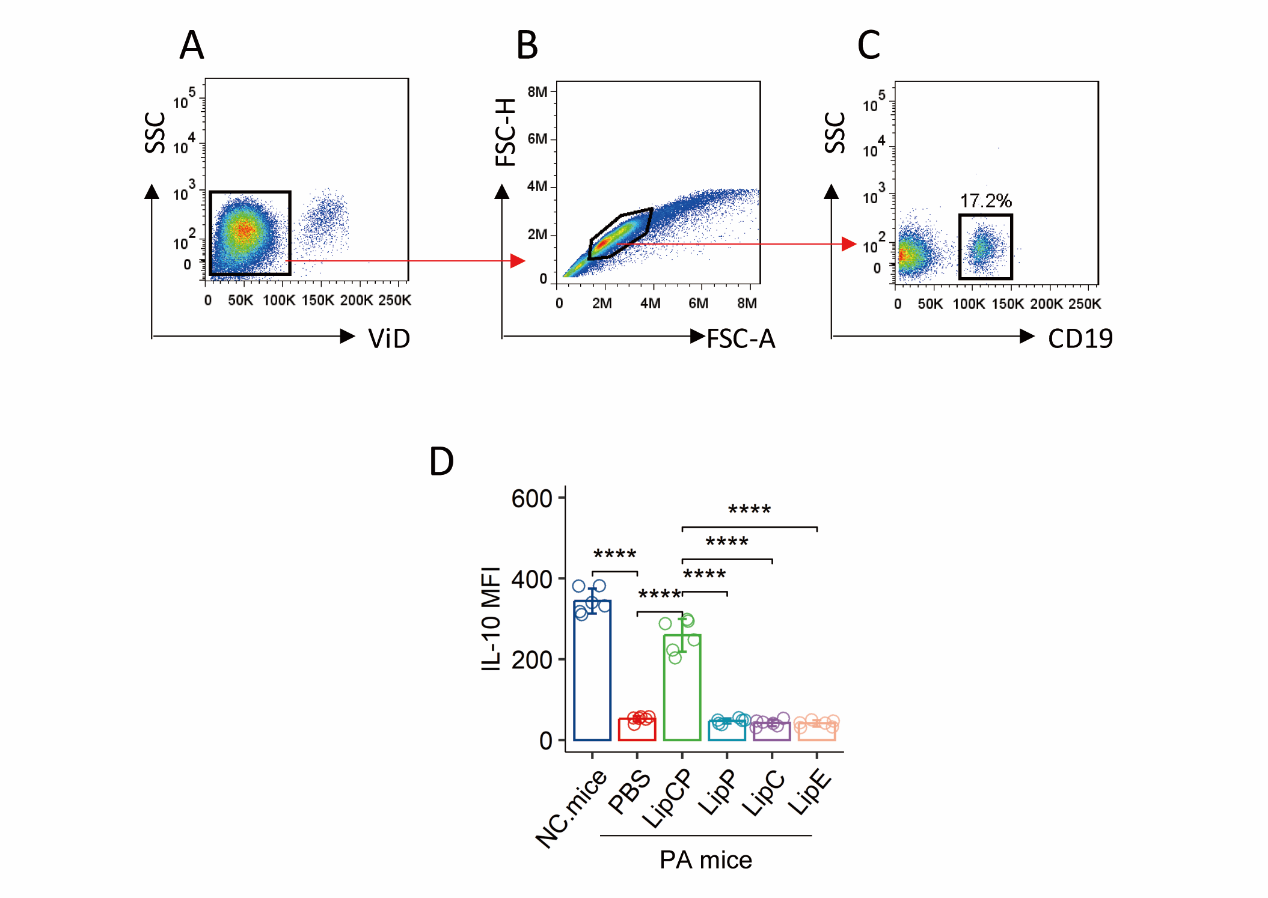


**Figure S2. Gating strategy of B cells**. LPMCs were prepared and analyzed by FCM. A, dead cells were gated out. B, adherent cells were gated out. C, CD19^+^ B cells were gated.

D, a supplemental figure to Fig. 3 in the main text. Bars show the levels of IL-10 MFI (mean fluorescence intensity) in Tr1 cells. ****p<0.0001 (One-way ANOVA with Tukey post hoc test). Each dot in bars presents one sample.


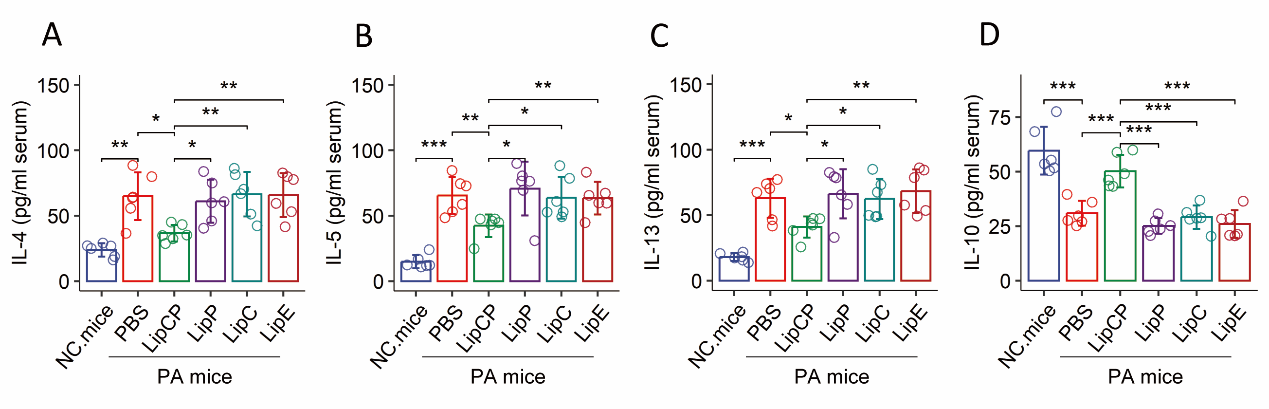


**Figure S3. Serum levels of Th2 cytokines (IL-4, IL-5, IL-13) and regulatory cytokine IL-10 in mice.**

Peanut allergy (PA) mice were treated with the indicated interventions (X-axis labels); detailed treatment protocols are provided in Fig. 1 and Methods.
A–D: Bar graphs depict serum levels of the Th2 cytokines IL-4 (A), IL-5 (B), and IL-13 (C), as well as the regulatory cytokine IL-10 (D), in mice.
Abbreviations: NC, naïve control; FA, food allergy; LipCP, liposomes co-loaded with CpG and peanut allergen; LipP, liposomes loaded with peanut allergen; LipC, liposomes loaded with CpG; LipE, empty liposomes.
Data are presented as mean ± standard deviation (SD); each dot represents an individual mouse sample.
Statistical analysis: One-way analysis of variance (ANOVA) followed by Tukey’s honest significant difference (HSD) post hoc test. Significance: *p<0.05, **p<0.01, ***p<0.001.


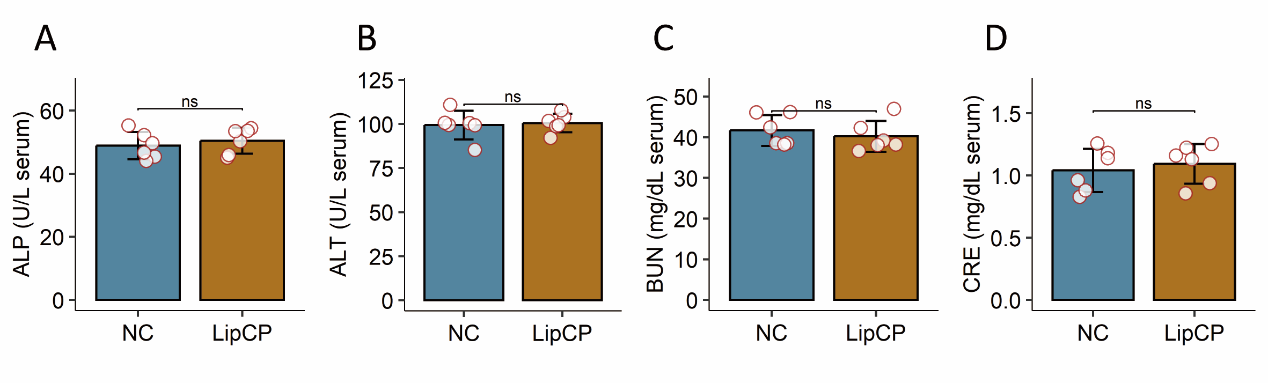


**Figure S4. Assessment of systemic toxic effects of LipCP**. Blood samples were collected from normal control mice and LipCP-treated mice, and serum was analyzed via enzyme-linked immunosorbent assay (ELISA). (A–D) Bar graphs depict serum levels of the indicated biochemical markers: ALP (Alkaline Phosphatase), ALT (Alanine Aminotransferase), BUN (Blood Urea Nitrogen), and CRE (Creatinine). ns: not significant (t test).


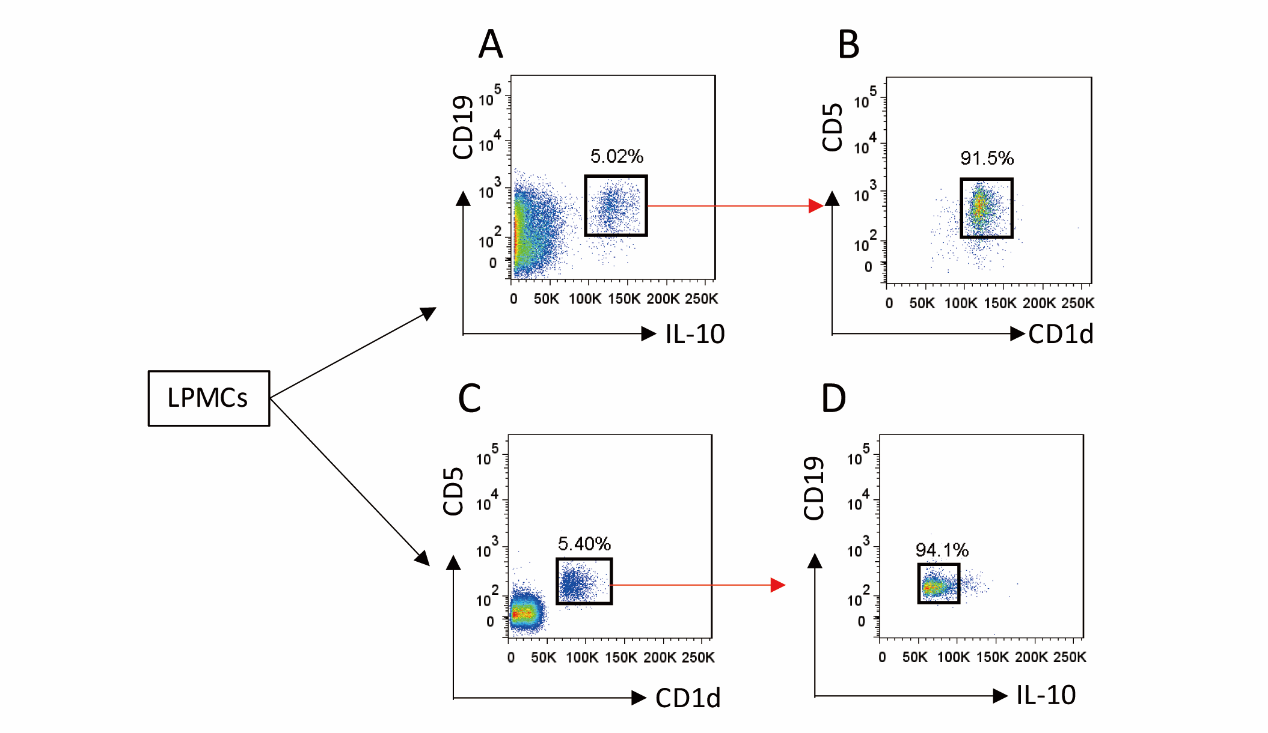


**Figure S5. Assessment of IL-10^+^MHC II^+^ DCs and CD1d^+^CD5^+^ DCs**. A, B10 cells were gated. B, B10 cells are also CD1d^+^CD5^+^. C, CD1d^+^CD5^+^ cells were gated. D, CD1d^+^CD5^+^ cells are also IL-10^+^CD19^+^.
